# Effect of MoO_3_ content on structural, physical and electrical properties of mixed alkali calcium phosphate glasses for energy storage capacitors

**DOI:** 10.1039/d5ra08604c

**Published:** 2025-12-12

**Authors:** A. G. Darwish, Mohamed I. Farouk, Mostafa I. Abdelglil, H. A. Abo-Mosallam

**Affiliations:** a Department of Microwave Physics and Dielectrics, Physics Research Institute, National Research Centre 33 El Buhouth St., Dokki Cairo 12622 Egypt abdelfatah.nrc@gmail.com ag.darwish@nrc.sci.eg; b Civil Engineering Department, College of Engineering, Imam Mohammad Ibn Saud Islamic University (IMSIU) Riyadh 11432 Saudi Arabia; c Colleges of Medical Technical, Al-Farahidi University Baghdad 00964 Iraq; d Glass Research Department, National Research Centre El-Buhouth St, Dokki Cairo 12622 Egypt abomosallamnrc@gmail.com ha.ebrahim@nrc.sci.eg

## Abstract

This work was motivated by the limited stability and dielectric performance of phosphate glasses. The main goal is to examine how small MoO_3_ additions (0–4 mol%) affect structure, elastic properties, and dielectric response in a mixed-alkali calcium phosphate host. New mixed alkali calcium phosphate with different MoO_3_ contents with nominal composition 10K_2_O–10Na_2_O–(20 − *X*)CaO–*X*MoO_3_–60P_2_O_5_ (mole%) glasses (where *X* = 0, 1, 2, and 4 mol%) were swimmingly prepared *via* melt quenching. The impact of doping with MoO_3_ on the structure, physical and mechanical strength of the prepared glass samples was studied. It is determined that MoO_3_/CaO molar ratio substitution led to an increase the density and oxygen packing density (OPD) and a decrease molar volume because the internal structure has changed for the non-crystalline specimens. The calculated mechanical parameters revealed that MoO_3_-modified samples have the highest mechanical resistance due to the increased bond strength and structural rigidity. The permittivity of glass samples was measured from 0.1 Hz to 10 MHz at temperatures from 25 °C to 150 °C. The neat glass showed little change in high-frequency permittivity. Adding MoO_3_ increased low-frequency permittivity and widened the dispersion range. Two processes appeared: a slow interfacial response at low frequencies and a faster dipolar response at mid-frequencies. The physical, mechanical and electrical data propose that the vitreous materials under study are varied conductors with both electronic and ionic conductivity as suitable for high-performance energy storage capacitor materials. The prepared glass is lead-free and composed of abundant, low-toxicity oxides, aligning with environmentally responsible and sustainable dielectric design.

## Abstract 2

Enhanced electrical transport in mixed-alkali calcium phosphate glasses: MoO_3_ doping at 1–2 mol% yields optimum conductivity *via* hopping pathways and network modification.

## Graphical abstract

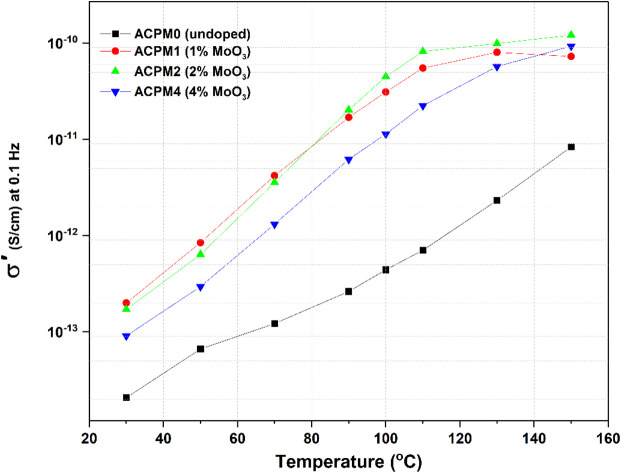

## 1 Introduction

Materials science is an interdisciplinary field that studies the properties, structure, and processing of materials to design and discover new ones for various applications. It combines principles from physics, chemistry, and engineering to solve problems across industries like aerospace, energy, medicine, and electronics. Selecting materials is crucial in engineering design for a sustainable and successful product development.^1–5^ Material performance, including mechanical, physical, and chemical, is crucial for meeting client expectations.^1–5^ Glasses are a unique range of ceramic materials defined principally by their atomic structure and have a highly disordered amorphous structure. Many current glasses still use heavy metal oxides like PbO or CdO based dielectric glasses, which raises environmental and processing concerns. This motivates for the development of glass compositions based on abundant and low-toxicity oxide networks. Mixed-alkali Ca-phosphate glasses offer such a route, as they are lead-free, rely on widely available elements, and allow controlled incorporation of transition-metal oxides to tailor dielectric behavior. The goal is to make glass compositions without these oxides while keeping good dielectric and mechanical properties. This supports the development of safer and more sustainable materials for clean energy use. Glasses based on the phosphate system are an interesting material with a variety of uses in energy storage, photonics, and optics applications. Because of its unique and innovative qualities and ability to maintain its glassy character, phosphate glass is the most widely used of the many diverse glass-forming ingredients.^6–8^ Low softening and melting points combined with high thermal expansion coefficient values are further characteristics of phosphate glasses. Understanding the fundamental ideas describing the local structures of the phosphate glasses on various distance regimes is necessary to enhance their properties with respect to future application.^6–8^ The addition of modifiers such as alkali and alkaline earth metals to the glass network changes the internal structure, which also affects the properties of the glass.^9,10^ Alkali metal-augmented phosphate glasses are classified as fast-ion-conducting (FIC) materials because they frequently show strong electrical conductivity. Because of their advantageous conductivity, these glasses are used as cathodes or electrolytes in batteries, among other uses.^11^ Their distinct physical characteristics include a high free volume and a lack of long-range order.^12^ They are useful in technologies that need electrical conductivity and effective ion transport for a range of commercial and scientific applications because of these characteristics. Many chemical, physical, and electrical properties, such as ionic conductivity, dielectric constant, and dielectric losses, change when multiple alkali compounds are added to glasses due to the mixed alkali effect.^13^

Transition-metal oxide (TMO)-containing phosphate glasses are appealing for a variety of electrochemical applications and other devices because they provide distinct conductivity types based on their composition as well as the content and function of TMO in the glass structure.^14^ The flexible valency of molybdenum ions as TMO helps to create a strong network within the glass, making molybdenum trioxide (MoO_3_) very beneficial in terms of mechanical strength, refractive index, and transparency.^15^ Consequently, the potential of MoO_3_-modified glasses in advanced optical and energy storage applications has drawn attention.^16^ In addition to being a conditional glass-forming chemical, molybdenum oxide has the ability to modify networks. This behavior results from the possibility of molybdenum in glass existing in several oxidation states. MoO_3_ participates in the glassy network through its connections by bridging oxygen atoms to create tetrahedral [MoO_4_] and octahedra [MoO_6_] in MoO_3_ rich areas.^9,10^ Conversely, the Mo^5+^ ion creates the structural unit [Mo^5+^O_3_]^−^, which functions as a network modifier.^17,18^ Molybdenum–phosphate glasses of various compositions have been explored recently; their electrical and ionizing transport capabilities make them a promising material for practical applications.^19–21^ Previous studies on MoO_3_–phosphate glasses have mainly focused on binary or ternary systems with single alkali modifiers, where Mo enters the network as MoO_4_ or MoO_6_ units and alters optical or dielectric properties. Other studies on mixed-alkali phosphate glasses have concentrated on the mixed-alkali effect and its influence on ionic transport. However, systematic work combining MoO_3_ substitution with a mixed-alkali calcium phosphate host, and correlating the resulting structural, mechanical, and dielectric changes, has been limited. The present study addresses this gap by investigating MoO_3_ additions in a Na_2_O–K_2_O–CaO–P_2_O_5_ system and linking the observed physical and electrical properties to both Mo coordination and mixed-alkali network effects. Consequently, the goal of the study is to synthesize new MoO_3_ doped alkali mixed calcium phosphate glasses based on the 10NaO–10K_2_O–(20 − *X*)CaO–*X*MoO_3_–60P_2_O_5_ composition (whereas *X* = 0, 1.0, 2.0, and 4.0 mol%), and to inspect the impact of MoO_3_ on the changed structure, physical mechanical and electrical properties.

## 2 Experimental techniques and theoretical calculations

### 2.1 Glass synthesizes

Using the traditional melting method, four samples of the 10K_2_O–10Na_2_O–(20 − *X*)CaO–*X*MoO_3_–60P_2_O_5_ (mol%) glass were prepared (where *X* = 0, 1, 2, and 4 mol%). These non-crystalline materials exact chemical compositions are listed in Table 1. The specimens were coded as ACPM0, ACPM1, ACPM2, and ACPM4 based on the MoO_3_ content. Components of the starting materials of K_2_CO_3_, Na_2_CO_3_, CaCO_3_, NH_4_H_2_PO_4_, and MoO_3_ were analar grade reagents used for the preparation of vitreous samples. After well-mixing for the glass batches, 50 grams of powder was melted in Al_2_O_3_ crucibles for 100 minutes at temperatures ranging from 1250 °C to 1300 °C. To cast and shape the solidified glass, the resultant melt was moved to a hot stainless steel plate. After 60 minutes at 400 °C in another muffle furnace, the synthesized samples were allowed to cool gradually to ambient temperature in order to remove the inner stress and strain.

**Table 1:** The composition and calculated parameters for the investigated glasses

Sample ID	K_2_O	Na_2_O	CaO	MoO_3_	P_2_O_5_	Density	Molar volume (*V*_m_)	Oxygen packing density (OPD)
ACPM0	10	10	20	0	60	2.51	44.15	74.73669583
ACPM 1	10	10	19	1	60	2.54	43.639	75.16156399
ACPM 2	10	10	18	2	60	2.56	43.304	75.28019528
ACPM 4	10	10	16	4	60	2.62	42.324	76.07858104

### 2.2 Materials characterization

Each synthesized sample was subjected to an X-ray diffractometer (PW3040/60, PANalytical, Netherlands) in order to confirm the vitreous irregular nature. The Cu-K_α_ radiation source was used to do the measurements at two theta with a scanning speed of 5° min^−1^ and a range of between 2*θ* = 5–80°. Different crystalline phases are identified by applying the PCPDF win software analysis. The FTIR absorbance spectra of the vitreous specimens were examined at room temperature in the 400–2000 cm^−1^ wave number range with the instrument resolution of 4 cm^−1^ using a spectrometer of type (PerkinElmer Instrument Co. USA), in order to determine the internal structural features of the non-crystalline materials. For the FTIR test, each glass sample was ground, mixed with KBr powder at a fixed mass ratio of 1 : 100, and then the obtained mixture was pressed into a thin disk by using a hydraulic tablet press under 1.5 ton of force. The density (*ρ*), molar volume (*V*_m_), oxygen packing density (OPD), and Oxygen molar volume (OMV) of the generated amorphous samples were measured in this investigation and entered into Table 1. The *ρ*, *V*_m_, OPD, and OMV values were calculated depending on eqn (1)–(4).^22–25^1
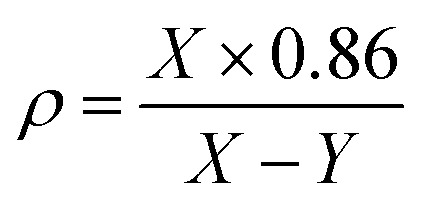
where *X* = weight of the sample measured in air. *Y* = weight of the specimen measured in xylene (density of xylene is 0.86 g cm^−3^).2
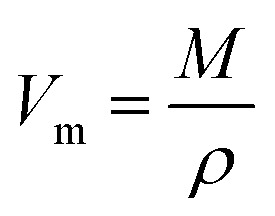
where *M* = average molecular weight of the glass and *ρ* = density.3
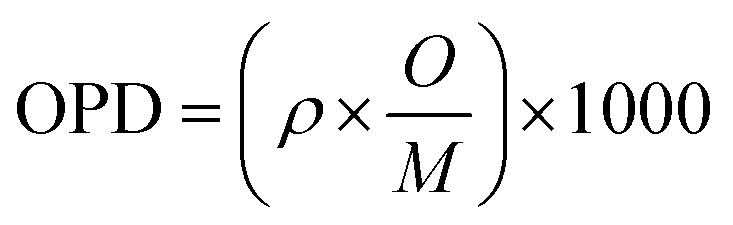
where ‘*O*’ is number of oxygen atoms in each oxide per unit formula, *M* is molecular weight of the glass sample

4 OMV = (∑*X*_i_*M*_i_/*ρ*)(∑1/*x*_i_*n*_i_)

where *X*_i_ = molar fraction of each component ‘i’ and *M*_i_ = molecular weight, *n*_i_ number of oxygen atoms in each constituent oxide.

### 2.3 Theoretical elastic modulus parameters

The synthesized specimens' elastic modulus was designed to theoretically extend the Makishima–McKenzie model.^26,27^ Using relations 5 and 6, the mechanical characteristics for the glass system oxides were determined based on the ionic packing density (*V*_t_) and the total dissociation energy per unit volume (*G*_t_).The following is how the elastic parameters are measured in connection with eqn (7) through (11).^26,27^5
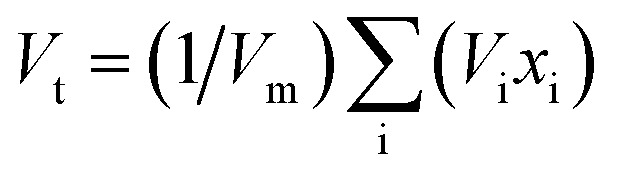
6
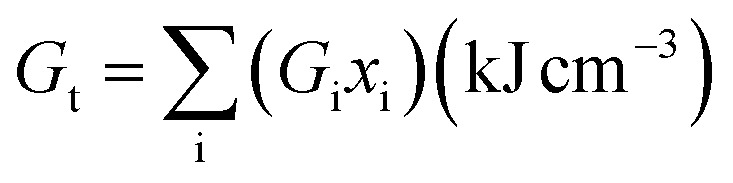
where, (*V*_i_) is the packing density factor and (*G*_i_) is the dissociation energy per unit volume of the contributed glass systems oxides.^28–30^

7 *E* = 1.2*V*_t_*G*_t_

8 *B* = 1.2*V*_t_*E*

9 *S* = 3*EB*/(9*B* − *E*)

10 *L* = *B* + (¾)*S*

11 *σ* = 0.5 − (*V*_t_/17.2)

here *E* is Young's modulus (GPa), *V*_t_ is the ionic packing density (dimensionless), and *G*_t_ is the total dissociation energy per unit volume (kJ cm^−3^). *B* is the bulk modulus (GPa), *S* is the shear modulus (GPa), *L* is the longitudinal modulus (GPa). *σ* is Poisson's ratio (dimensionless). Eqn (7)–(11) are derived from the Makishima–Mackenzie model^26,28,31,32^ for estimating the theoretical elastic moduli of oxide glasses. The model links mechanical properties to two structural descriptors: ionic packing density (*V*_t_), which reflects how tightly the atoms are packed, and dissociation energy per unit volume (*G*_t_), which reflects bond strength. Using these parameters, the model estimates Young's modulus, bulk modulus, shear modulus, longitudinal modulus, and Poisson's ratio, providing a theoretical basis for comparing compositions.

• The Young's modulus (*E*), also known as the elastic modulus, quantifies the inherent rigidity of a material and its ability to withstand deformation when subjected to an applied force.

• The shear modulus (*S*), is a physical property that quantifies a material's ability to resist deformation when subjected to a shear stress, which is a force that induces the sliding of adjacent layers inside the material.

• The bulk modulus (*B*) is a physical property that quantifies the ability of a material to withstand uniform compression or expansion.

• The longitudinal modulus (*L*) is a measure of a material's stiffness, representing the ratio of longitudinal stress to longitudinal strain under uniaxial stress.

### 2.4 Electrical properties

The dielectric measurements were done by an Alpha-A analyzer of a Broadband Dielectric Spectroscopy system (Concept 40, Novocontrol Technologies) in the frequency span 0.1 Hz to 10 MHz, and temperatures span of 30 to 150 in 8 steps. The electrical measurements in this work were carried out in a solid-state dielectric configuration. Rectangular glass plates (∼10–12 mm length, ∼1.5–2 mm thickness) were polished for parallelism, and silver paste was applied on opposite faces as electrodes to ensure good electrical contact.

## 3 Results and discussion

### 3.1 Structural properties

#### 3.1.1 Amorphous nature of the ACPM materials

**Fig. 1:**
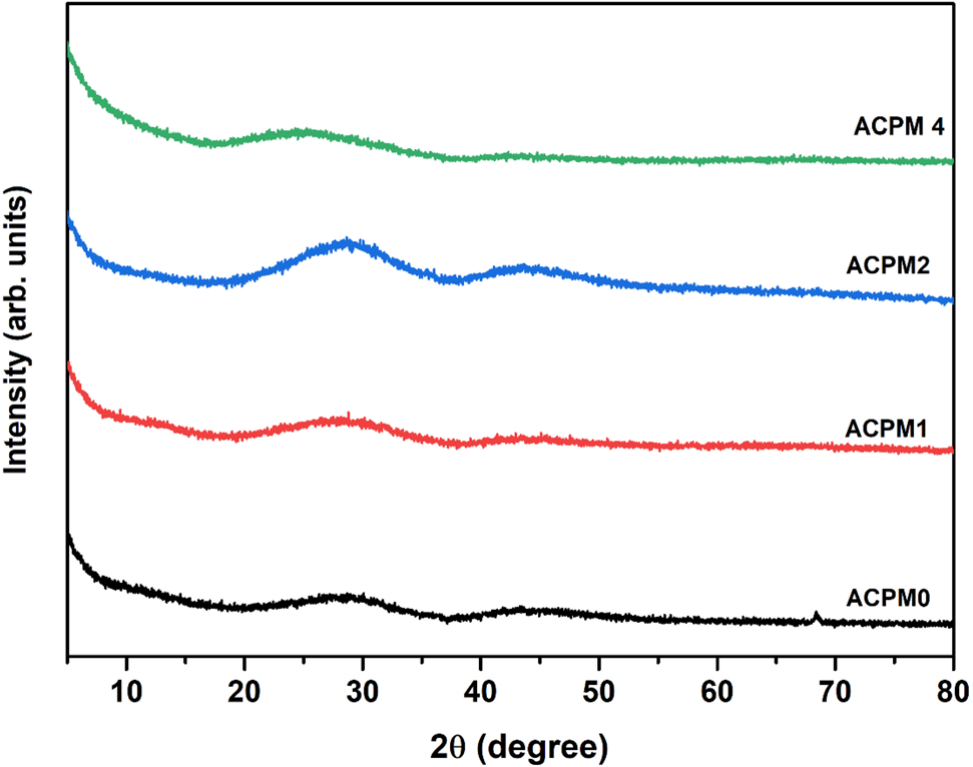
XRD patterns for all glass samples APCM0, APCM0, APCM0, and APCM4, modified with MoO_3_ at 0, 1, 2, and 4 mol%, respectively.

The obtained XRD patterns for the manufactured materials with a 2*θ*° between (5–80°) are shown in Fig. 1. The absence of prominent peaks indicates that the materials created are completely non-crystalline. The typical broad barrow at 2*θ*° between (20–35°) confirms the amorphous and glass-like nature of the specimens being analyzed.^33,34^

#### 3.1.2 FTIR analysis

**Fig. 2:**
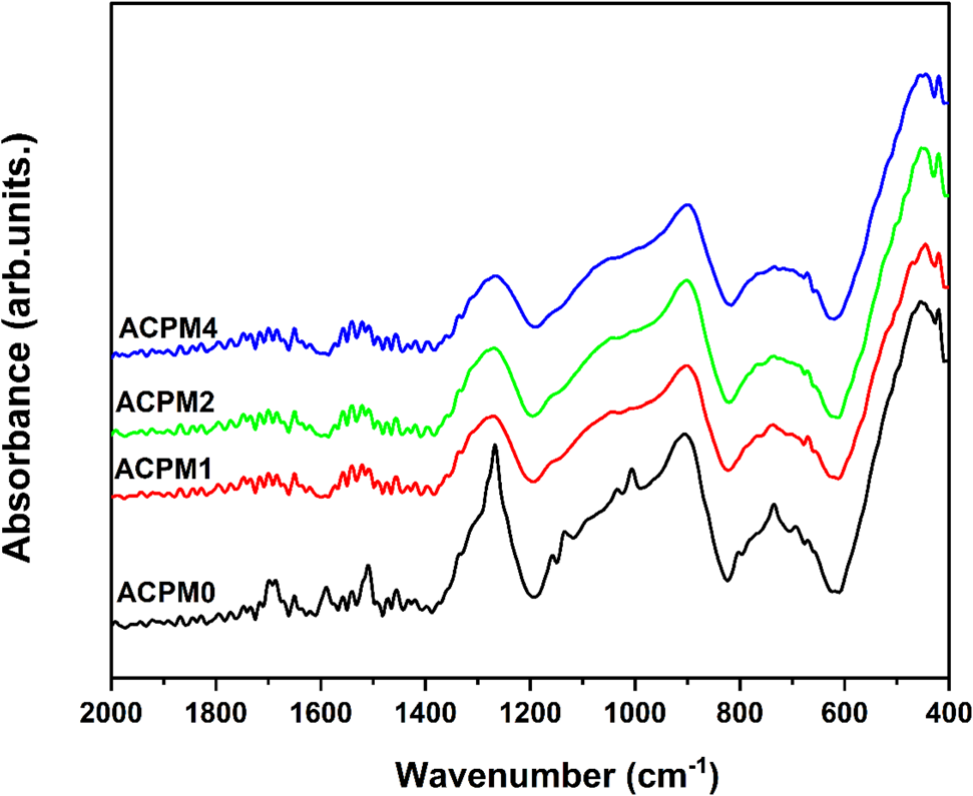
FTIR absorbance spectra for all glass samples APCM0, APCM1, APCM2, and APCM4, modified with MoO_3_ at 0, 1, 2, and 4 mol%, respectively.

FTIR, or Fourier transform infrared spectroscopy, is a crucial method for identifying modifications to the internal structure of glass materials. The change in the internal structure of the glass, which is reflected in the change in the peak positions appearing on the FTIR spectrum, is due to changes in the type quantity and nature of chemical bonds as well as the strength of the glassy lattice connectivity.^35^ Modified oxides in the glass network play a major and important role in changing the internal structure by breaking bonds and forming bridge bonds such as M–O–PO_3_ (where M = K^+^, Na^+^ cations). The effects of doping alkali calcium phosphate glass with MoO_3_ on the changes of FTIR absorption spectra in the region of 400–1600 cm^−1^ is shown in Fig. 2. It is noticed that the spectra of all non-crystalline specimens contain four broad bands. The absorption spectrum detected in the base glass ACPM0 wavenumber range 400–600 cm^−1^ at 600, 545 and 455 cm^−1^ are due to the bending vibrations of O=P–O and/or P–O–P bonds.^36^ The absorption bands at about 741 and 669 cm^−1^ are attributed to the symmetric stretching vibrations of P–O–P bonds.^36,37^ The absorption band appeared at 902 cm^−1^ and 1064 cm^−1^ is due to the vibration of symmetric stretching of PO_3_ groups characteristic of Q1 structural units and asymmetric stretching vibrations of (PO_3_) chain terminal groups respectively.^36,38,39^ The spectrum at 1273 cm^−1^ is assigned to the asymmetric stretching vibration of, (PO_2_) as, the two non-bridging oxygens linked to phosphorus atom in Q2 tetrahedral structural units.^40,41^ With the addition MoO_3_ to the glass matrix it is possible ions to penetrate in the network a former or a modifier as MoO_4_ or MoO_6_ units respectively. Developing of new links between MoO_6_ and the different network components formed causes a rise in network connectivity and recommends a rigidly packed amorphous composition.^42^ The asymmetric stretching modes of PO_3_^2−^ group from Q1 units give rise to a band centered at ∼1116 cm^−1^, while both asymmetric stretching vibration of P–O and PO_2_^−^ modes from Q2 units are responsible for the weak absorption shoulder from ∼1276 cm^−1^.^43,44^ Thus, according to the FTIR results, the glass matrix has the local structure mainly based on pyro- and metaphosphate units (Q1 and Q2, respectively) with a small contribution of tetra- and triborate units. The essential change detected in the FTIR spectra by the replacement of 1, 3 and 5 mol% of calcium by molybdenum in the glasses is related to the shift in the location of the band about 1273 cm^−1^. Whereas the band at ∼1650 cm^−1^ may be attributed to O–H bending vibrations. The addition of MoO_3_ led to an increase in the phosphate network polymerization and long phosphate chains.^45^ The addition of MoO_3_ in the glass matrix composition caused a shift in the position of absorption band at 1266 cm^−1^ as a consequence of its broadening. No vibration of the Mo–O linkages was detected in the infrared spectral range.^44^ These results are in complete agreement with previous results in which molybdenum oxide was added up to 7 mol to the phosphate glass based on the system P_2_O_5_–CaO–B_2_O_3_–K_2_O– and no peaks attributed to MoO_3_ appeared in the FTIR results.^44^

### 3.2 Physical parameters [density, molar volume, OPD, OMV]

**Fig. 3:**
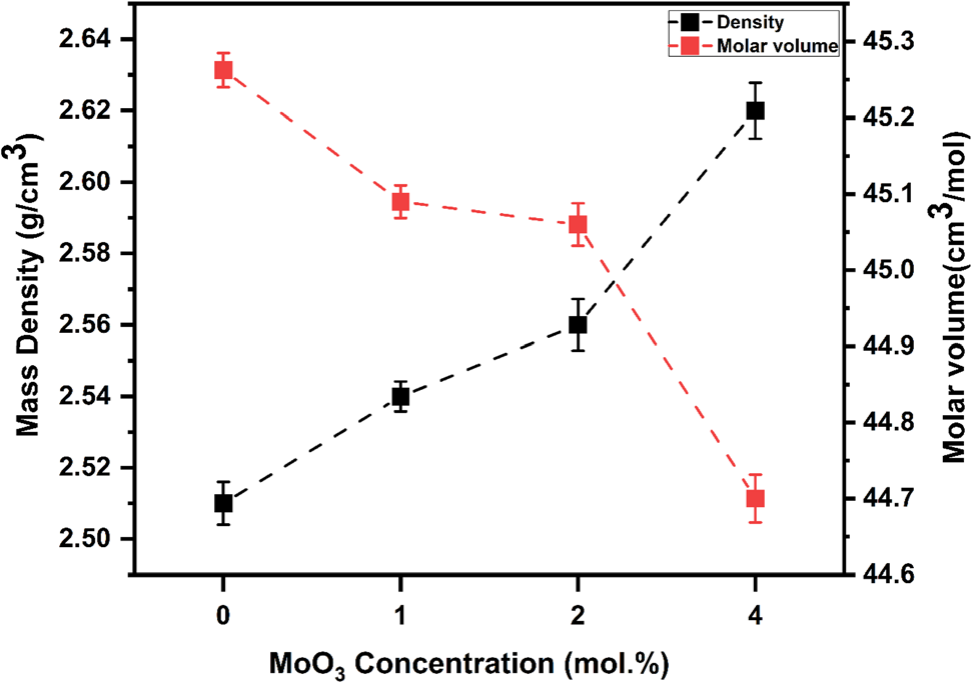
The densities (*ρ*) and molar volumes (*V*_m_) for all glass samples APCM0, APCM0, APCM0, and APCM4, modified with MoO_3_ at 0, 1, 2, and 4 mol%, respectively.

**Fig. 4:**
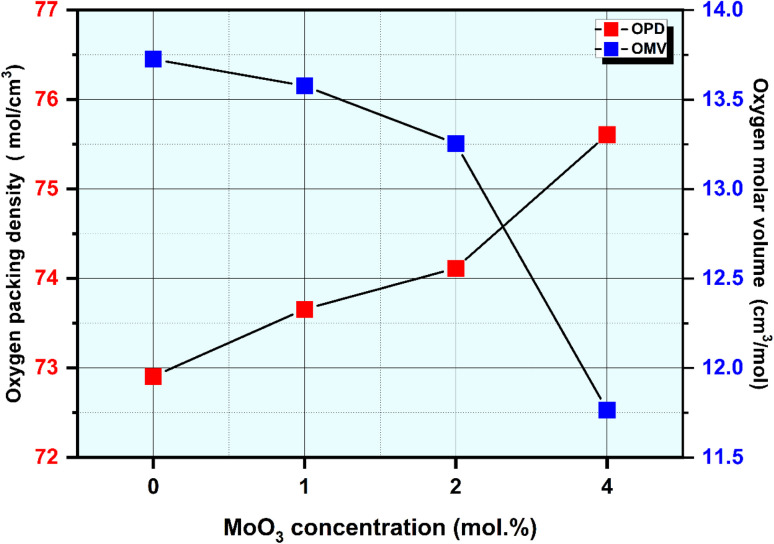
The OPD and OMV for all glass samples *vs.* MoO_3_ level.

The experimentally determined density *ρ* and molar volume *V*_m_ of the produced amorphous materials with different MoO_3_ content are displayed in Fig. 3. The findings demonstrate that as the MoO_3_ content rises, the materials' density rises from 2.51 to 2.64 g cm^−3^. The motive for the linear rise in density in produced materials with MoO_3_ doping could be that MoO_3_ has a larger density and atomic weight (*ρ* = 9.64 g cm^−3^ and *M*_w_ = 143.94 g mol^−1^) than CaO (*ρ* = 3.34 g cm^−3^ and *M*_w_ = 56.0774 g mol^−1^), respectively. These results indicate that the glass construction is denser as a result of the addition of molybdenum oxide. Molar volume, *V*_m_, measures the specific volume occupied by 1 g of oxygen atoms and is particularly crucial to the connectivity of the glass. According to the synthesized non-crystalline specimens *ρ* and *M*_w_, it was possible to define the molar volume, where it is decreased over the interval of 45.262–44.703 cm^3^ mol^−1^ when the MoO_3_ content is enhanced to 4 mol% as shown in Fig. 3. Because MoO_3_ ions function as network modifiers and occupy spaces inside the glass, resulting in a more compact arrangement with higher density and reduced *V*_m_ values, the results show that *V*_m_ values gradually drop with increasing MoO_3_ content.^46^ Because MoO_3_ has a smaller ionic size than calcium (Mo^6+^: 0.065 nm, and Ca^2+^: 0.098 nm), adding molybdenum oxide to glass materials instead of calcium oxide frequently results in a drop in molar volume.^15^ An indicator of the degree of compactness and arrangement of oxygen atoms in treated glasses, the oxygen packing density (OPD) quantifies how well these atoms are packed inside the glass network.^47^ The oxide system's rigidity, on the other hand, is indicated by the volume pulse.^47^Fig. 4 demonstrates OPD and OMV against MoO_3_ content in mol%. The results also showed that increasing the molybdenum content of the glass samples led to a decline in the oxygen molar volume (OMV) from 13.725 to 11.764 cm^3^ mol^−1^, and increase oxygen packing density (OPD) from 72.907 to 75.608 g atm per L. This pattern implies that as the MoO_3_ content rises, the precursor glasses become more stiff and compact. Network connectivity increases as new connections between MoO_6_ and the various network components created in the glass are developed.^42^ The production of bridging oxygen and the disappearance of NBO atoms could be another reason for the deteriorating OMV parameters.^42^

### 3.3 Mechanical parameters

**Fig. 5:**
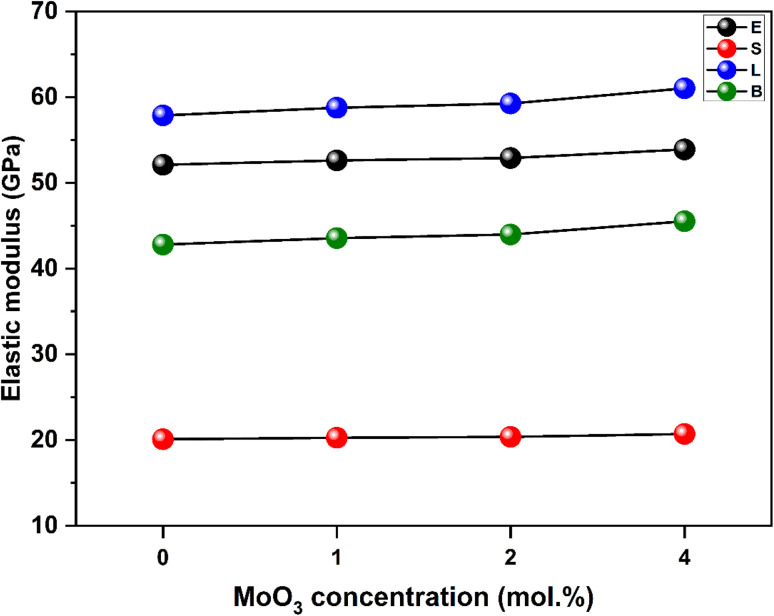
The estimated mechanical characteristics, including the longitudinal (*L*), bulk (*B*), shear (*S*), and Young's (*E*) moduli, *vs.* MoO_3_ level.

**Fig. 6:**
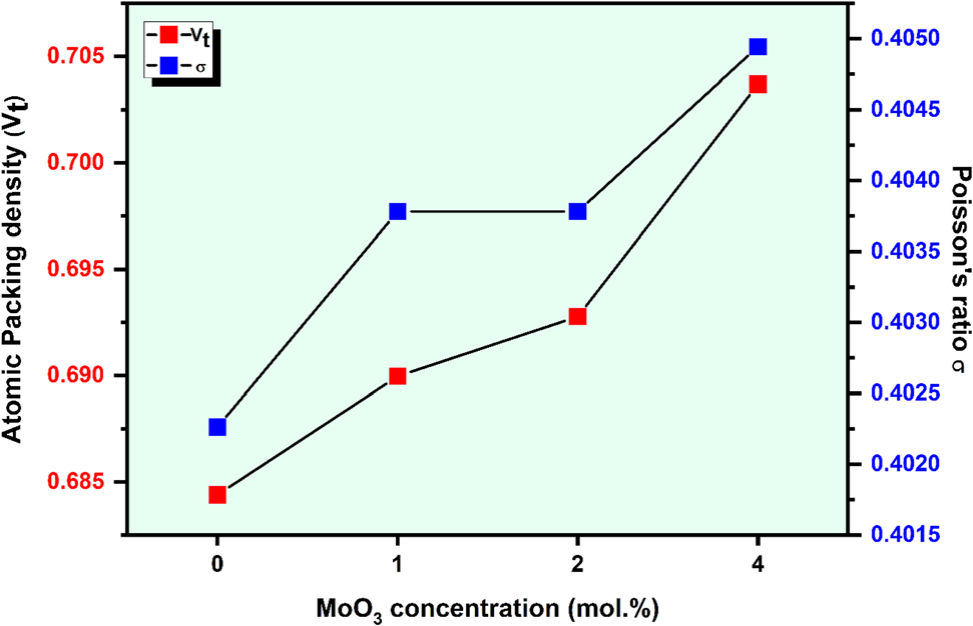
The atomic packing density (*V*_t_) and Poisson ratio (*σ*) *vs.* MoO_3_ level.

The well-known Makishima–Mackenzie model was used to calculate the elastic characteristics of synthesized glasses. The dissociation energy (*G*_t_) and packing density parameter (*V*_t_) values were determined to calculate the different elastic parameters such as Young's modulus, *E*; bulk modulus, *B*; shear modulus, *S*; longitudinal modulus, *L*; and, Poisson's ratio, *σ* as summarized in Table 2 and graphically represented in Fig. 5 and 6. The results showed that the (*G*_t_) decreased from 38.070 × 10^6^ (kJ m^−3^) for ACPM0 to 38.292 × 10^6^ (kJ m^−3^) for ACPM4 prepared specimens. However, the (*V*_t_) of the ACPM-glasses diverse from 0.6844 (m^3^ mol^−1^) for ACPM0 glass specimen to 0.7037 (m^3^ mol^−1^) for ACPM4 glass specimen. The calculated mechanical parameters of the prepared non-crystalline specimens showed varying values, as follows: Young's modulus (*E*) miscellaneous between 52.110 and 53.901 GPa, the bulk modulus (*B*) miscellaneous between 42.797 and 45.516 GPa, the shear modulus (*S*) miscellaneous between 20.088 and 20.689 GPa, the longitudinal modulus (*L*) miscellaneous between 57.863 and 61.033 GPa and from 0.404 to 0.402 for (*σ*). The increase in the calculated elastic modulus (*Y*_m_, *E*_m_, *S*_m_, *L*_m_) with doping MoO_3_ in the glasses based on alkali calcium phosphate can be attributed to increasing the structural rigidity and compactness of the glasses. Owing to the fact that the small ionic size of Mo^6+^ is 0.065 nm in comparison to that of Ca^2+^ at 0.098 nm, the glass structure converts to be much more compact.^15,48^ The results are fully consistent with the experimental and theoretical elastic modulus results of previous studies, which indicated that the elastic modulus increases with increasing MoO_3_ content in the glass due to the higher connection strength of MoO_6_ groups.^42^ As the concentration of MoO_3_ increases in the prepared glasses elastic modulus gradually increases. The reason for this tendency is the increased intrinsic modulus of MoO_3_ (110 GPa), which reinforces the overall network structure. Molybdenum trioxide, being a transition metal oxide, typically enhances the stiffness and strength of glass matrices by increasing cross-linking within the glass network. MoO_3_ serves as a network modifier and can partially occupy interstitial sites within the glass framework, improving the rigidity of the entire arrangement.^49^ The progressive increase in MoO_3_ content thus results in an enhanced elastic modulus due to the greater mechanical stiffness provided by this oxide.^49^ The rise in elastic moduli showed that the elasticity of glasses had been enhanced by the addition of MoO_3_.^50^ The decrease in non-bridging oxygens (NBOs) caused by the increase in MoO3 content replacing other oxides may be the cause of the slight rising elastic moduli. As a result of the improved stiffness of the glass network and the decline in NBOs.^50^

**Table 2:** The elastic moduli parameters for the investigated glasses

	Young's modulus (*E*)	Longitudinal modulus (*L*)	Shear modulus (*S*)	Bulk modulus (*B*)	Poisson's ratio (*σ*)
ACPM0	52.11022	20.08775	57.86289	42.79708	0.40226
ACPM1	52.6138	20.25629	58.75455	43.56233	0.40378
ACPM2	52.90597	20.356	59.24919	43.98219	0.40378
ACPM4	53.90067	20.68916	61.03281	45.51594	0.40494

## 4 Dielectric study

### 4.1 The permittivity (*ε*′) analysis

**Fig. 7:**
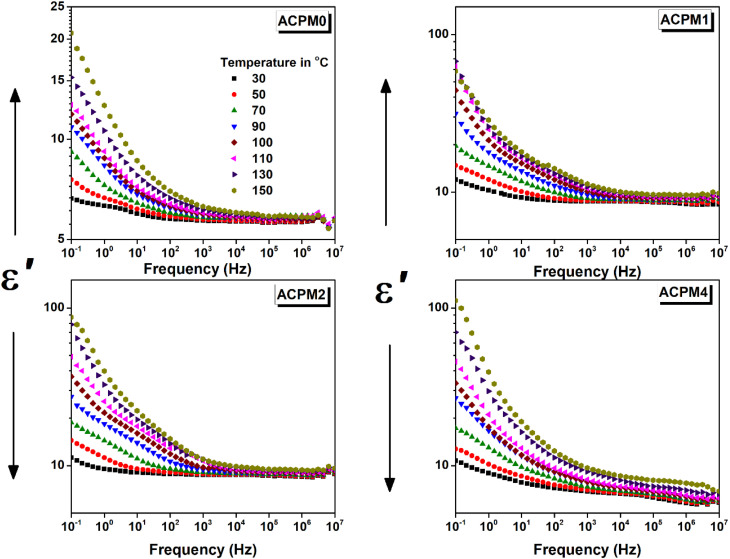
The permittivity (*ε*′) *vs.* frequency for MoO_3_ modified glasses, at temperatures between 30 and 150 °C with step 20 °C as indicated.

**Fig. 8:**
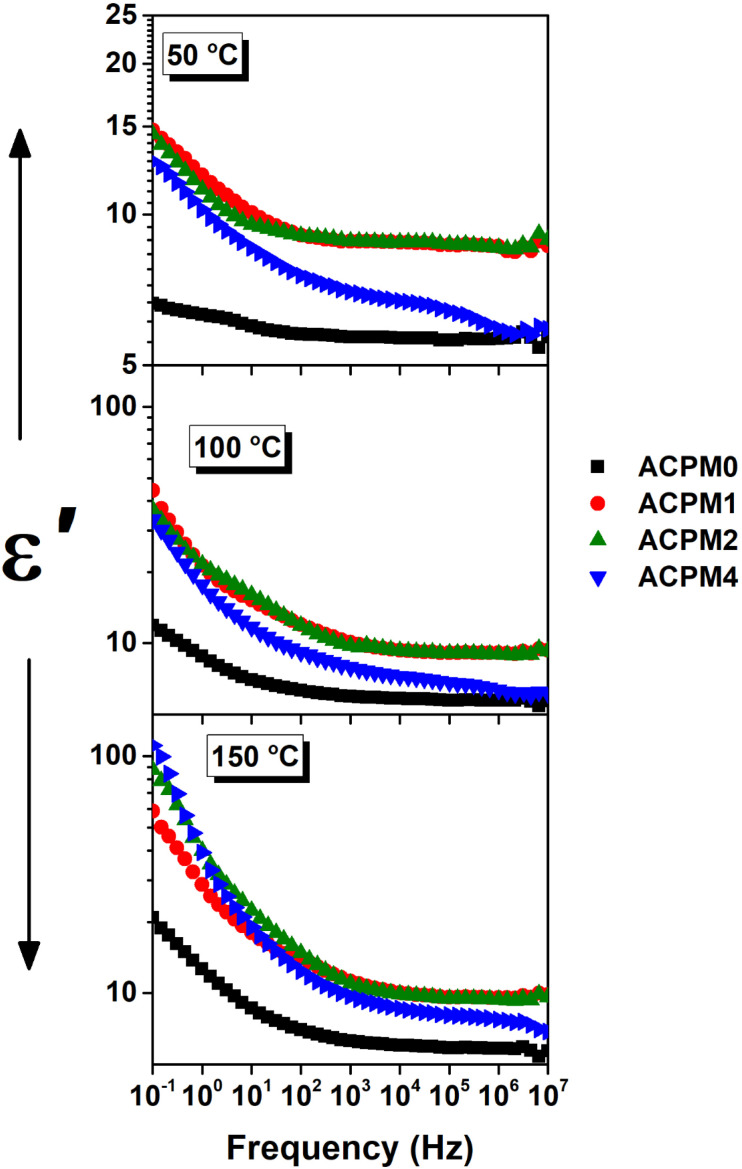
The permittivity (*ε*′) *vs.* frequency for all samples at three selected temperatures, 50, 100, and 150 °C.

The permittivity (*ε*′) trends *vs.* frequency for all samples at varied temperatures is presented in Fig. 7. The permittivity shows typical dispersion behavior at low-frequency then sits at frequency independent plateau formed at high-frequency.^51^ For the sample ACPM0, *ε*′ decreases with frequency across all temperatures. At 25 °C, *ε*′ decreases from 6.6 at 0.1 Hz and sits at around 5.6 in a plateau extending from 100 Hz to 10 MHz. At 150 °C, *ε*′ falls from 20.8 at 0.1 Hz and saturates at around 5.8 in a plateau extending from 21.5 kHz to 10 MHz. The dispersion region in fact is composed of two parts of different drop rates. The drop is sharp at low-frequency and then slows down in mid-frequencies. The temperature rise mainly affects low-frequency *ε*′, while high-frequency *ε*′ is barely changed or it remains mostly stable. All the curves seem to level from about 100 kHz and above. The sample ACPM1 shows stronger temperature dependence of *ε*′, especially at low frequencies. At 0.1 Hz *ε*′ increases from 12 at 25 °C to 67 at 150 °C, and at 100 kHz it increases from 8.5 at 25 °C to 9.6 at 150 °C. At 150 °C, *ε*′ falls from 20.8 at 0.1 Hz and saturates at around 5.8 in a plateau extending from 21.5 kHz to 10 MHz. High-frequency *ε*′ stabilizes at different levels depending on temperature but ranges between ∼8.5–9.5. Compared to ACPM0, the frequency dispersion is broader and steeper, also a greater contrast is noticed between low and high-frequency permittivity. The temperature effect fades at higher frequencies and the low-frequency range seems more sensitive. The sample ACPM2 shows further permittivity increment at higher temperatures. At 150 °C and 0.1 Hz, *ε*′ starts at 87 then falls and saturates as 9.5, while at 25 °C, it decreases from 11.2 at 0.1 Hz then sits at 8.7. The permittivity changes seem non-monotonic with doping. Even the dispersion span changes are nonsystematic, the dispersion in ACPM1 is wider than that of ACPM2. Temperature rise leads to significant improvement in *ε*′, especially below 1 kHz, and slope of the dispersion curve is steeper than ACPM0 and ACPM1. The sample ACPM4 shows best temperature enhancement, even at high frequency we notice clear separation between the curves relative to the other samples. The permittivity values of ACPM4 at 0.1 Hz changes between 10.7 and 111.2 at 25 and 150 °C, respectively. While at 100 kHz *ε*′ changes from 6.2 to 8.1 at 25 and 150 °C, respectively. The frequency dispersion of ACPM4 shows steepest frequency drop, with broad relaxation span. Temperature effect is obvious all over the frequency span, but it shows more influence below 1 kHz. The plateau levels are temperature dependent, yet the values of *ε*′ seems lower than other samples at low temperature. For a clearer picture of that non-monotonic behavior, the permittivity for all samples are plotted together *vs.* frequency at three selected temperatures as presented in Fig. 8.

Comparing all samples' permittivity trends at 50 °C we notice the following. The permittivity values of ACPM1 and ACPM2 are close with dominance of ACPM1. The MoO_3_ doping seems to increase the permittivity at low concentration, but then the permittivity relapse and decrease. However, the permittivity values of the doped samples remain higher than the neat sample. The permittivity values order as ACPM1 > ACPM2 > ACPM4 > ACPM0. The plateau of the neat sample spans more than other samples, while spanning least and showing two step dispersion of different slopes and they nearly take up most of the frequency spectrum. At 100 and 150 °C, ACPM0 continue with the least value and shows wider flattened curves. ACPM4 2nd drop becomes smoother and its plateau grows wider. The values of ACPM1 still higher than ACPM2 by small fraction, and ACPM4 shows higher values below 10 Hz and at 150 °C. The plateau of ACPM2 spans less at 100 and 150 °C, and its dispersion range increases. The dispersions at low frequency for all samples gets sharper drop with heating.

The very steep drop at low frequencies points to slow interfacial or space-charge effects.^52^ The second, gentler decline from ∼100 Hz up to ∼10 kHz suggests a separate, faster process that most likely dipolar reorientation or short-range ionic hopping.^53^ At high frequencies, there is intrinsic electronic polarization limit so a plateau forms at ∼100 kHz and above. The sample ACPM0 has few polarizable centers and weak interfacial effects, which shows narrow as small *ε*′ values spanning between ∼6.6 → 20.8, along with small plateau shift. ACPM1 has wider *ε*′ span (∼12 → 67) reflecting added polarizable MoO_4_-related dipoles at low MoO_3_ content. ACPM2 permittivity at 150 °C (∼87) exceeds ACPM1's (∼67), showing continued rise in polarizable centers at 2 mol% MoO_3_. ACPM4 shows the steepest *ε*′ drop and the largest temperature-driven boost (10.7 → 111.2) indicating maximal interfacial/dipolar activity from high density of slow polarization centers and pointing to broad relaxation-time distribution caused by extensive MoO_3_-induced network modifications. The strong upward shift of permittivity curves with temperature at low frequencies indicates thermally activated dipole reorientation or hopping. At high frequencies, this temperature dependence becomes weaker and the shift with temperature is much smaller. ACPM0–ACPM2 plateaus barely move. ACPM4's plateaus move more with heat. This change shows that fast processes work the same at all temperatures while slow processes need heat. Fast polarization modes show little change with temperature, while slow polarization modes need heat to activate. At low frequencies, slow modes dominate and shift up strongly with temperature. At high frequencies, fast modes dominate and the permittivity stays nearly the same. ACPM4's high-frequency plateau still contains some slower polarization contributions. The MoO_3_-rich network in ACPM4 spreads its relaxation times to higher frequencies.^53^ As temperature rises, those slower modes shift upward into the plateau region. That makes ACPM4's plateau moves more with heat, even at high frequencies. The permittivity plateaus ordering (ACPM2 > ACPM4 > ACPM1 > ACPM0) implies incremental rise in intrinsic polarizability up to 2 mol% MoO_3_ then slightly drops at 4 mol%. The dispersion-steepness ordering (ACPM4 > ACPM2 > ACPM1 > ACPM0) reflects density of slow polarization centers and breadth of relaxation-time spectrum. A steeper fall means more slow polarization modes spread over a wider frequency range, which is consistent with a denser distribution of slow relaxers (dipoles, space charge regions) in the more heavily doped glasses.

XRD curves show amorphous patterns ensuring no crystalline pathways, so polarization arises from glass network and interfaces.^54^ FTIR rise of Q^2^ band (at 1273 cm^−1^) with MoO_3_ confirms increased bridging oxygens, leading to stiffer network that narrows low-frequency permittivity span in ACPM2 → ACPM4.^53^ A stiffer network traps and immobilizes interfacial and dipolar sites that caused large low-frequency *ε*′ in the less-rigid samples. Moving from ACPM1 to ACPM4, those slow polarization centers shrink in number or mobility, so the low-frequency permittivity span narrows. The absence of new sharp IR bands matching continued broad *ε*′ dispersion, indicating overlapping MoO_4_ and MoO_6_ units rather than discrete new modes.^53^ The fact that no new sharp bands appear with adding MoO_3_ means that the Mo-related vibrations sit under, or merge into, the existing broad phosphate bands. Tetrahedral MoO_4_ and octahedral MoO_6_ units both get absorbed in regions already crowded by P–O–P and O=P–O bands. As MoO_3_ increases, no clean or separate peak for MoO_6_ or for MoO_4_, instead, those Mo vibrations simply broaden and slightly shift. Because the IR modes for MoO_4_ and MoO_6_ overlap with each other and with phosphate modes, this ends up with a continuous distribution of local environments rather than distinct species. This matches the appearance of the permittivity-frequency behavior as a broad, smooth dispersion without sharp steps. Each small change in local Mo coordination adds or shifts a bit of polarizability over a wide frequency range. The result is a non-Debye, overlapping relaxations spectrum instead of discrete, narrow relaxations. Another evidences of the preceding claims comes from noticing the physical properties of the samples. The density increase (2.51 → 2.64 g cm^−3^) and molar-volume decrease (45.26 → 44.70 cm^3^ mol^−1^) correlates with reduced free volume, narrowing dispersion window.^55^ OPD rise (72.9 → 75.6 g atm per L) and elastic-moduli increase (*E*, *B*, *S*, *L* all ↑) linking to network rigidification that begins to suppress polarizability above ∼2 mol% MoO_3_,^56^ explaining ACPM2 non-monotonic *ε*′ and ACPM4 plateau-shift behavior. Density rises and molar volume falls with more MoO_3_, oxygen-packing density and all elastic moduli goes up. These trends correlate to a stiffer, and more cross-linked network. A stiffer network traps slow polarization centers and spreads their relaxation times more widely. That explains why ACPM4 shows the steepest *ε*′ drop and the broadest dispersion span. For ACPM0–ACPM2, the high-frequency *ε*′ plateau hardly shifts with temperature. That matches a tighter network with fewer slow dipoles or interfacial charges slipping into the fast-mode region. ACPM4's plateau moves more on heating because its broad relaxation-time distribution pushes some slower modes into the high-frequency window.

### 4.2 The dielectric loss (*ε*′′) analysis

**Fig. 9:**
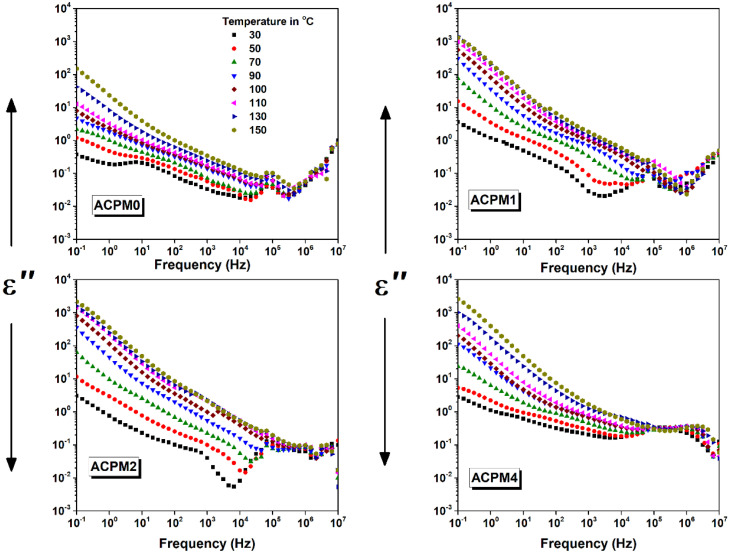
The dielectric loss (*ε*′′) *vs.* frequency for MoO_3_ modified glasses, at temperatures between 30 and 150 °C with step 20 °C as indicated.

The dielectric loss (*ε*″) of all the samples plotted *vs.* frequency and temperature using same ranges employed in the permittivity analysis, as shown in Fig. 9. The loss starts in a decreasing manner from low frequencies, then a broad shoulder forms below ∼10 Hz, and at higher frequency a pump or a peak is noticed. The shoulder is obvious in all samples, but with more clarity at 30 °C in sample ACPM0, then it starts to fade with either adding MoO_3_ or with heating. The high frequency bump shows most prominent presence in sample ACPM4, also ACPM4 shows larger loss amplitudes ACPM4. Both of the two features moves to higher frequency by the increase in either of MoO_3_ content or temperature. At higher temperature the low-frequency shoulder almost disappears, while the bump shows its prominent shifts with temperature at higher MoO_3_ content.

The loss falls at the lowest frequencies because very slow polarization can follow the field and then saturates.^57^ A broad shoulder appears below ∼10 Hz when interfacial or space-charge polarization can no longer keep up and begins to lag. The main peak at higher frequency marks the dipolar relaxation where permanent or induced dipoles reorient most effectively.^58^ Each feature shifts to higher frequency with more MoO_3_ or higher temperature because those processes speed up under thermal activation or added polarizable units. The low-frequency shoulder fade with MoO_3_ content and heating due to charge transport masking other dynamics. Heating and MoO_3_ rising conductivity and fewer slow interfacial sites mask the slowest polarization processes at low frequency whereas faster dipolar relaxations still stand out. ACPM4 has the highest density of dipolar or hopping relaxation centers, so the mid-frequency bump most pronounced in it. Both of the shoulder and the bump shift to higher frequency with temperature and MoO_3_ doping because thermal energy and extra Mo-related polarizable units speed up dipole reorientation and hopping, moving their characteristic frequency upward. The loss features broaden and overlap rather than form sharp peaks because a wide distribution of local environments creates non-Debye relaxations with smeared-out loss signatures. The shoulder fading with temperature and MoO_3_ doping matches reduced low-frequency permittivity span and weaker space-charge polarization in ACPM2 → ACPM4. The mid-frequency bump showing prominence in ACPM4 aligns with its steep second-region permittivity drop and broad relaxation-time distribution. The upward shift of shoulder/bump with temperature and MoO_3_ doping mirrors permittivity curves moving up and dispersion knees shifting to higher frequency. Larger loss amplitudes in ACPM4 reflect its strongest temperature-driven permittivity rise below ∼1 kHz. XRD shows all glasses are amorphous, so loss features come from glass network and interfaces. FTIR Q^2^ band (1273 cm^−1^) grows with MoO_3_, meaning more P–O–P bridges and fewer NBOs. Fewer NBOs and tighter network reduce slow interfacial polarization, making low-frequency shoulder fade in doped samples. The increased density and decreased *V*_m_ with MoO_3_ doping reduce free volume, shifting shoulder and bump to higher frequency. OPD increase signals more cross-links, which broadens relaxation-time distribution and makes loss peaks wider. Elastic moduli (*E*, *B*, *S*, *L*) all rise with MoO_3_ doping, showing stiffer network that spreads out dipolar relaxation frequencies, and shifts bump further. Mechanical stiffening traps some slow modes, so ACPM4 shows largest loss amplitude and strongest bump shift. The tighter network forces faster relaxations into mid-frequency region, matching shoulder and bump moving to higher frequency.^52^

### 4.3 The AC conductivity (*σ*′) analysis

**Fig. 10:**
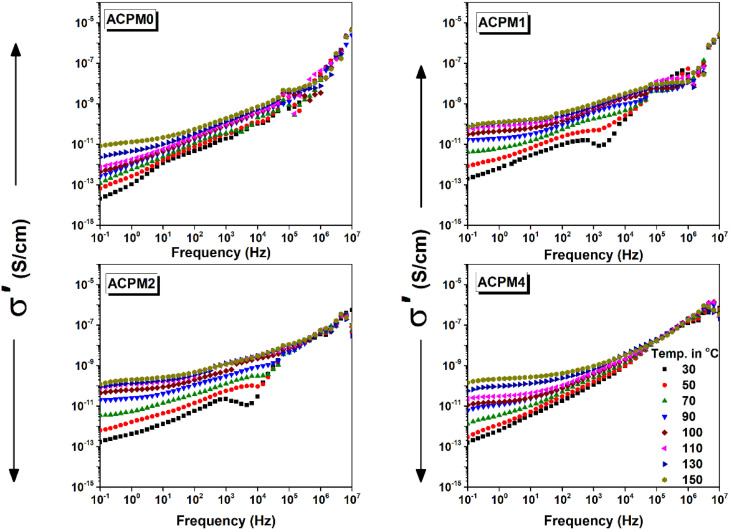
The AC conductivity (*σ*′) *vs.* frequency for MoO_3_ modified glasses, at temperatures between 30 and 150 °C with step 20 °C as indicated.

Fig. 10 presents AC conductivity (*σ*′) *vs.* frequency at different temperatures for all glass samples. The conductivity shows strong frequency dependence, especially at high frequencies, which indicates presence of multiple conduction mechanisms. At low frequencies, nearly flat or slowly rising *σ*′ plateau exists, indicating DC-like region. These regions correspond to DC conductivity plateau, where charge carriers can follow the field. At higher frequencies, the conductivity increases more steeply, indicating onset of frequency-dependent AC conductivity. This high-frequency upturn in *σ*′ is due to localized hopping or slow reorientation motion of dipoles that cannot follow the field fully. The transition from low-frequency plateau to dispersive region shifts toward higher frequencies with increasing temperature, because the heating led to faster charge dynamics and reduced relaxation times.^51^ For all samples, *σ*′ increases with temperature at every frequency, which indicates thermally activated conduction mechanism, likely small polaron hopping. Thermal activation causes the conductivity to rise gradually, and it is more noticeable at lower frequencies. At higher temperatures (≥110 °C), *σ*′ increases notably at low and mid frequencies. At temperatures of 130–150 °C, dispersion slope becomes steeper, especially in doped samples, which indicates enhanced high-frequency polarization and rapid short-range ionic hopping. The heating improves the conductivity by about 2 and half orders for the neat sample, and by about 3 orders for all the doped samples, and they almost have the same values at 0.1 Hz. The doping itself increases the conductivity only by one order for all samples. MoO_3_ doping seems to enhance thermal activation effects. Sample ACPM0 conductivity starts gradual increase with frequency then sharp frequency dependence. Almost no clear plateau until very high temperature, which is due to limited free carrier contribution at low temperatures. Two peak-like features appear and seem to correlate with those noticed in the loss curves. They are possibly related to dipolar relaxations or interfacial polarization. The two features move forward with heating, which is a signature of thermally activated dynamics, nonetheless, they fade gradually, especially the one at low-frequency, which is likely related to large-scale charge accumulation or Maxwell–Wagner-type interfacial polarization which dissipates at higher temperatures. The sample conductivity values are lowest among all samples, due to the absence of MoO_3_ which reduces mobile charge carrier density compared to doped samples. Sample ACPM1 has low-frequency plateau slightly elevated compared to ACPM0, beside conductivity values start higher than ACPM0, which is due to increased mobile ion contribution due to MoO_3_ presence. Frequency at onset of dispersion shifts slightly right with increasing temperature. Conductivity curves are less dense and spread out compared to ACPM0. The bumps are apparent but the transition from the one at low frequency is followed by a dip. The feature at low-frequency is partially shifted to higher frequency compared with that noticed in ACPM0. The slight rightward shift in dispersion onset and the low-frequency bump with temperature due to decreased relaxation time, relaxation becomes faster with MoO_3_-doping. The dip after low-frequency feature before high-frequency rise relates to separation of conduction processes or overlapping mechanisms. The low frequency region shows slow growth with frequency at temperatures 30 and 50 °C, then a plateau forms at 70 °C and above. The heating above 70 °C causes clear plateau formation, where thermal energy enables DC conduction dominance over localized polarization. The plateau span gets wider with heating. Sample ACPM2 show similar behavior by forming plateau 70 °C and above, and it expands with temperature indicating improved free carrier contribution. It also has a dip between the two features, but at frequencies above 100 kHz the conductivity curves converge at all temperatures. This convergence above 100 kHz implies common high-frequency conduction behavior regardless of temperature, possibly due to fast localized motions dominating. Sample ACPM4 (with the highest MoO_3_ content) doesn't show plateau until 90 °C, which may be caused by strong localized polarization or ion blocking at low temperature. Also the low frequency feature is not apparent, while the gentle bump or curvature at high frequencies is still observable above 100 kHz. The low-frequency feature is suppressed which may indicate reduced large-scale charge buildup due to increased percolation pathways, the glass gains many more local, AC-active centers without necessarily boosting its long-range DC transport. The curvature at high frequencies is visible and becomes dominant, which represents fast hopping conductivity or small-polaron type behavior.

The permittivity showed low-frequency dispersion and flattening at high-frequency, which mirrors AC conductivity where plateau forms at low-frequency and dispersion follows. The low-frequency shoulder in *ε*″ matches region where *σ*′ shows gradual rise and both related to interfacial polarization. The loss peak in mid-to-high-frequency aligns with bump/curvature in *σ*′, where both arise from dipolar or ionic relaxation. The shift of *ε*″ peaks with MoO_3_-doping and temperature parallels the rightward movement of *σ*′ features, as relaxation processes are thermally and structurally modulated. The fading of low-frequency *ε*″ feature at high temperature mirrors disappearance of low-frequency shoulder in *σ*′ where conduction masks polarization loss. The samples with no clear *ε*′ plateau also lack *σ*′ plateau, and both signals suppressed free carrier transport. The real permittivity and conductivity trends are consistent, higher MoO_3_ samples show lower *ε*′ but higher *σ*′ as increased conduction suppresses storage but enhances loss. The emergence of dips between peaks in *ε*″ and *σ*′ relates to overlapping or competitive dielectric relaxation processes.

All glasses show amorphous XRD patterns, so charge moves through the disordered network. Young's, bulk, shear, and longitudinal moduli all go up. A tighter, stiffer network leaves less free volume for long-range ion motion. That pushes the low-frequency conductivity plateau to higher temperatures and narrows its width. The dispersive, frequency-dependent region also shifts to higher frequencies. ACPM4, with the highest density and modulus, shows the smallest DC-like plateau and the earliest, steepest AC rise, matching its most rigid structure.

**Fig. 11:**
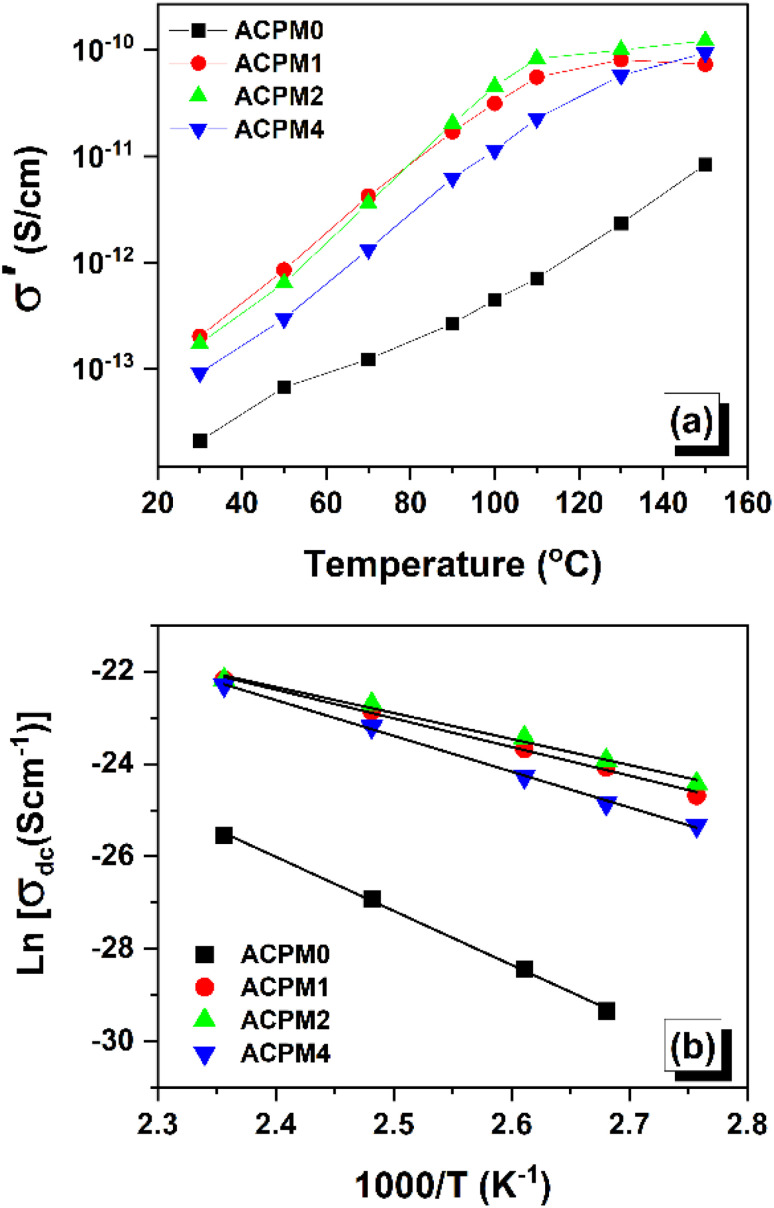
(a) The AC conductivity (*σ*′) *vs.* temperature for MoO_3_ modified glasses, at fixed frequency 0.1 Hz, (b) Arrhenius plots used to extract activation energy (*E*_a_) from ln(*σ*_dc_) *vs.* 1000/*T* for the glass samples.

The conductivity of the samples at 0.1 Hz *vs.* temperature is demonstrated in Fig. 11(a). Conductivity at 0.1 Hz increases with temperature for all samples. Heating raises conductivity by roughly 2.5 orders for ACPM0 and about 3 orders for the Mo-doped samples. The doped glasses conduct much better than the undoped glass across the whole temperature range. At every temperature, ACPM2 and ACPM1 show the highest conductivities. ACPM2 (2 mol% MoO_3_) is the best performer. ACPM1 closely follows ACPM2. ACPM4 gives higher conductivity than ACPM0 but lower than ACPM1/ACPM2. The clear separation between doped and undoped samples confirms that MoO_3_ addition introduces more mobile carriers. ACPM0 shows low values at low temperatures, indicating poor charge mobility in the absence of Mo. The conductivity growth rate is steeper for the doped samples, showing stronger thermal activation of carriers. The convergence of ACPM1 and ACPM2 at high temperature suggests that both compositions reach a similar maximum conduction level when thermal energy is sufficient. ACPM4 rises with temperature but never reaches ACPM1 or ACPM2, suggesting limited long-range pathways compared to the optimal compositions. The smooth curves without sudden anomalies indicate stable conduction mechanisms across the measured temperature range. Overall, the 1–2 mol% MoO_3_ range provides the best conductivity improvement, while higher MoO_3_ content enhances conductivity but not beyond the optimal level.

The activation energies for the samples were estimated using Arrhenius relation, *σ* = *σ*_0_ e^−*E*_a_/*kT*^, where *E*_a_ is the activation energy, *T* is the absolute temperature, *σ*_0_ is a pre-exponential constant, and *k* is the Boltzmann constant. The DC conductivity values were extracted from plateaus of AC conductivity curves following Jonscher's equation fit, then plotted *vs.* 1000/T as demonstrated in Fig. 11(b).

The activation energy shows a U-like behaviour with minimum at MoO_3_ content = 1–2 mol% (lowest at 2%). ACPM1 and ACPM2 shows lowest activation barrier and best polaronic conduction (small-polaron hopping/short-range hopping). ACPM0 has network-limited ionic or high-barrier baseline transport in undoped glass. ACPM4 is an intermediate case with more local hopping centers but reduced long-range connectivity. The samples with low *E*_a_ show clearer DC plateau at lower temperatures and larger conductivity increase with heating. This matches lower activation barriers. ACPM0 has lowest *σ*′ and needs higher temperature to show DC-like behavior, which is consistent with its high *E*_a_. ACPM4 shows delayed DC plateau and strong high-frequency rise. Its *E*_a_ (0.67 eV) is higher than the optimal window but lower than undoped, which is consistent with many local AC-active centers but poorer long-range hopping. There are structural indicators that relates to this behavior. In FTIR, the Q^2^ band grows with Mo addition, which means more P–O–P bridges and fewer NBOs that tighten the network. The increased density oxygen packing and moduli, and reduced molar volume with Mo addition, all of that suggests a tighter, and stiffer network with reduced free volume and long-range ion motion. This explains why *E*_a_ rises again at high MoO_3_ content. Small MoO_3_ additions creates hopping sites and lower *E*_a_, while excess MoO_3_ densifies the network, hinders hopping pathways and raises *E*_a_. Undoped glass lacks mixed-valence centers, so *E*_a_ remains high ( as shown in Table 3).

**Table 3:** The activation energy for the investigated glass samples

	ACPM0	ACPM1	ACPM2	ACPM4
Activation energy (*E*_a_)/eV	1.008	0.535	0.486	0.668

The addition of MoO_3_ to phosphate glass boosts its low-frequency permittivity and broadens its relaxation spectrum, while network stiffening controls high-frequency behavior.^51^ The doped samples show strong temperature dependence of permittivity, which points to thermally activated dipole and ion dynamics. The high and tunable permittivity values and the broad relaxation spectrum make these glasses good for high-temperature capacitors, tunable dielectrics in sensors, devices requiring broad-band dielectric tuning, and energy-storage materials.^59^

## 5 Limitations and future work

The present work is limited to 0–4 mol% MoO_3_, chosen to capture initial structural and dielectric changes while avoiding devitrification and processing issues at higher loadings. Future work will extend the series to 0.5–10 mol% with DSC/XRD screening, Raman/XPS for Mo coordination, and replicate melts to confirm whether the 1–2 mol% permittivity feature is robust. The present work focuses on overall conductivity trends, while more detailed separation of ionic *versus* electronic contributions remains a subject for follow-up. In future work we may extend the study by using blocking electrodes in impedance spectroscopy to distinguish ionic from electronic contributions, and by applying Nernst–Einstein analysis.

## 6 Conclusion

The structure, physical, mechanical and electrical properties of phosphate glasses in the K_2_O–Na_2_O–CaO–MoO_3_–P_2_O_5_ system have been examined. The physical parameters of the glasses measured indicated that replacing CaO with MoO_3_ led to an increase density, OPD with decreasing the molar volume, thereby improving the compactness of the synthesized amorphous materials. The calculated mechanical parameters confirmed that the incorporation of MoO meaningfully enhanced mechanical strength attributed to indicates enhanced network connectivity. The drop in permittivity became steeper in the order ACPM0, ACPM1, ACPM2, ACPM4. The ACPM4 sample showed the largest increase in low-frequency permittivity. The glass network became stiffer as density and moduli rose. That stiffening narrowed the low-frequency permittivity span. Dielectric loss features shifted to higher frequency with more MoO_3_ and higher temperature. AC conductivity showed a clear DC-like plateau and a dispersive region. Conductivity rose with temperature in all samples. Small-polaron hopping likely drives conduction. These results suggest that the lead-free, low-toxicity glasses show tunable dielectric properties and network stability. The MoO_3_ modified alkali phosphate glasses have potential as safe and sustainable choice for high-temperature capacitors.

## Conflicts of interest

The authors declare that they have no known competing financial interests or personal relationships that could have appeared to influence the work reported in this paper.

## Supplementary Material

RA-015-D5RA08604C-s001

## Data Availability

The data supporting this article have been included in the article, and will be available upon reasonable request. Supplementary information (SI) is available. See DOI: https://doi.org/10.1039/d5ra08604c.
